# Predictors at diagnosis for initiation of biologic or targeted synthetic disease-modifying anti-rheumatic drugs in patients with spondyloarthritis and psoriatic arthritis

**DOI:** 10.1186/s41927-026-00698-w

**Published:** 2026-09-29

**Authors:** Mohaned A. Hameed, Sofia Exarchou, Ankita Sharma, Ulf Bergström, Jon T. Einarsson, Carl Turesson

**Affiliations:** 1 Rheumatology, Department of Clinical Sciences, Malmö, Lund University, Malmö, Sweden; 2 Skåne University Hospital, Malmö, Sweden; 3 Rheumatology, Department of Clinical Sciences, Lund, Lund University, Lund, Sweden

**Keywords:** Psoriatic arthritis, Spondyloarthritis, Biologic DMARDs, Treatment initiation, Predictive factors, Bone marrow edema

## Abstract

**Background:**

There is limited data on factors predicting the initiation of biologic and targeted synthetic disease modifying anti-rheumatic drugs (bDMARDs/tsDMARDs) in Spondyloarthritis (SpA) and Psoriatic arthritis (PsA). The relative importance of objective inflammation and other patient factors in this context is unclear.

**Methods:**

A retrospective cohort study included consecutive patients diagnosed with SpA and PsA in 2012–2016 at a single rheumatology center. Data at diagnosis were obtained from medical records and from linkage to national and regional registers. Baseline predictors of b/tsDMARD initiation were assessed by Cox regression.

**Results:**

Among 119 PsA patients (mean age 49 years, 49% male), 41.7% initiated b/tsDMARDs (median 15 months after diagnosis). In patients with PsA, older age was negatively associated with b/tsDMARD initiation (hazard ratio (HR) per standard deviation 0.74; 95% confidence interval (CI) 0.55–1.00), but no other significant predictors were identified. Among 120 SpA patients (mean age 38 years, 64% male), 41.5% initiated bDMARDs (median 7 months after diagnosis). Predictors included elevated CRP levels (HR 2.68; 95% CI 1.44–5.01) and bone marrow edema (BME) on magnetic resonance imaging of sacroiliac joints (SIJ) (HR 1.94; 95% CI 1.02–3.70). There was no significant association with age at SpA diagnosis, or with sex, education, family background, country of birth, or comorbidities.

**Conclusions:**

Elevated CRP and SIJ BME at diagnosis were associated with bDMARD initiation in patients with SpA, while older patients with PsA may be less likely to start b/tsDMARDs. Education and ethnicity did not seem to influence b/tsDMARD initiation, suggesting equitable treatment access.

**Clinical trial number:**

Not applicable.

**Supplementary Information:**

The online version contains supplementary material available at https://doi.org/10.1186/s41927-026-00698-w.

## Introduction

In this era of rapidly advancing medical science, there has been a proliferation of novel medications for psoriatic arthritis (PsA) and spondyloarthritis (SpA). Patients with these chronic inflammatory conditions, which historically have been challenging to treat, now benefit from an expanding arsenal of disease-modifying antirheumatic drugs (DMARDs). Among biologic DMARDs (bDMARDs), tumor necrosis factor inhibitors (TNFi) and interleukin-17 (IL-17) inhibitors constitute effective therapies for both SpA and PsA, while interleukin-12/23 (IL-12/23) and interleukin-23 (IL-23) inhibitors have a treatment indication only for PsA. Alongside these, targeted synthetic DMARDs (tsDMARDs), such as Janus kinase (JAK) inhibitors, have emerged as effective therapeutic agents for PsA and SpA [1].

In the management of PsA and SpA, the primary goal is to achieve minimal disease activity, with an ideal outcome of remission or at least low disease activity [2–4]. To support this objective, the Treat-to-Target (T2T) strategy is recommended, involving proactive treatment adjustments to reach and maintain these clinical targets [2, 3]. The benefits of the T2T approach are partly attributed to an increased and timely use of bDMARDs

However, the direct costs of bDMARDs are substantially higher than those of csDMARDs. Consequently, variations in access to bDMARDs among patients with Inflammatory Joint Diseases (IJDs) exist across different nations due to budgetary constraints within healthcare systems as demonstrated by Putrik et al. in a questionnaire-based cross-sectional study in 49 European countries [2].

Reduced inequity and improved standard of care are important goals for evidence-based guidelines. Recommendations from international rheumatological societies, like the American College of Rheumatology (ACR) [3, 4], the Assessment of Spondyloarthritis international Society (ASAS) and European Alliance of Associations for Rheumatology (formerly European League Against Rheumatism, EULAR) [5, 6], as well as the national treatment guidelines from the Swedish Society of Rheumatology provide essential direction for treatment decisions, particularly in the early stages of PsA and SpA. All these recommendations/guidelines emphasize the importance of assessing disease activity and considering extra-musculoskeletal features in treatment selection. However, in addition to such parameters and healthcare system-related factors, clinical decision-making also involves a number of other items like patient-related factors (e.g., age, comorbidities, health literacy), treatment-related factors (e.g., prior medication use, potential adverse effects), and healthcare provider-related factors (e.g., treatment philosophy, familiarity with specific medications). Although various factors influencing treatment decisions have been identified, their relative importance in guiding clinical decision-making remains unclear. Identifying the key predictors of bDMARD or tsDMARD initiation in newly diagnosed PsA and SpA patients is critically important, particularly in the early stages of disease. Timely initiation of these therapies can be crucial in preventing disease progression, reducing irreversible joint damage, and ultimately improving long-term prognosis. Understanding the drivers of this decision process is essential to overcome current treatment gaps, such as delays in starting biologics, which often result in worse outcomes and compromised quality of life. The aim of this study was to investigate the relation for baseline characteristics at diagnosis (e.g. signs of disease severity) and patient background factors (e.g. demographics) with subsequent initiation of treatment with b/tsDMARDs in patients with newly diagnosed SpA and PsA.

## Methods

### Study design and participants

This retrospective cohort study investigated the relation between a number of clinical and sociodemographic factors at diagnosis and the initiation of b/tsDMARDs in patients diagnosed with SpA or PsA. The study was carried out at the outpatient rheumatology clinic of Skåne University Hospital in Malmö. All consecutive adult patients with a new registered diagnosis of SpA (ICD-10 codes M459, M460, M468 or M469) or PsA (ICD codes L405, M070, M071, M072, M073) in 2012–2016 were identified using the local patient administrative system. The diagnostic codes for SpA cover patients with both axial and peripheral SpA. The diagnosis was verified based on the expert opinion of a senior rheumatologist (CT) after a structured review of the medical records. The proportion of patients fulfilling the ASAS criteria for SpA and the CASPAR criteria for PsA using available clinical and imaging data was also evaluated [7, 8]. The phenotypic classification of SpA or PsA was based on the assessment of the predominant musculoskeletal manifestations by the managing physician and was verified by the expert opinion of two authors (MHA and CT). In one case, the diagnosis was changed by the authors from PsA to SpA, and in one case from SpA to PsA.[7, 8]

### Data collection

Clinical data from the visit when the diagnosis of SpA and PsA was first registered were extracted through structured reviews of the patients’ medical records. For patients with SpA, reports on magnetic resonance imaging (MRI) scans of the sacroiliac joint (SIJ) obtained around the time point of diagnosis (closest available, 88% within +/- 6 months) were evaluated for the presence or absence of bone marrow edema (BME), fatty lesions, and erosions. For patients with PsA, reports on plain radiographs (or x-rays) of the hands and feet obtained between three months prior to diagnosis and six months post-diagnosis were systematically reviewed for the presence or absence of juxta-articular erosions, arthritis-related joint space narrowing (JSN), and bone proliferation.

Additionally, treatment data were obtained from the Swedish National Quality Register (SRQ), and from complementary case record reviews, through December 31, 2021. The SRQ is a nationwide registry that collects data on patients with rheumatic diseases in Sweden. The main purpose of the SRQ is to improve the quality of care for patients with rheumatic diseases by providing healthcare professionals and researchers with comprehensive data for analysis and research purposes [9, 10].

Comorbidity data were retrieved from the regional healthcare register; the Skåne Healthcare Register (SHR). The Skåne Healthcare Register (SHR) was established in 1998 [11] and covers the utilization of both primary and secondary care services. This comprehensive approach allowed for a thorough evaluation of the prevalent comorbid conditions in the study population. Comorbidities were categorized into three distinct groups: those related to pain, psychiatric conditions, and other relevant health issues as previously described [12, 13] (Supplementary Table 1). A comorbidity was classified as prevalent if the corresponding ICD-10 code for the diagnosis was documented in the patient’s medical records prior to the diagnosis of SpA/PsA. Furthermore, the Charlson Comorbidity Index (CCI) a well-established tool to estimate the combined comorbidity burden, based on recorded diagnoses preceding the SpA/PsA diagnosis, was computed for each patient [14, 15].

In order to provide a broader context for understanding the diverse backgrounds and characteristics of the study participants, sociodemographic details, including level of formal education, country of birth, and family background, were derived from the Total Population Register held by Statistics Sweden [16]. Education level was categorized into three levels: lower secondary or less, upper secondary or short tertiary education, and bachelor’s degree or higher or equivalent. Family background was categorized as Swedish (both parents and the patient born in Sweden) or foreign (≥1 parent born abroad, irrespective of the patient s country of birth).

### Censoring and follow-up

Patients were followed from the time-point of the first SpA/PsA diagnosis registration to the initiation of a first bDMARD/tsDMARDs and were censored at the time of death, emigration from Sweden, or at the conclusion of the study on December 31, 2021. This approach ensured that the analysis considered relevant events and outcomes up to the specified endpoint.

### Statistical analyses

To investigate the relationship between patient characteristics at the time of diagnosis and the initiation of the first bDMARD/tsDMARD, Cox proportional hazard regression analyses evaluating the time to first bDMARD/tsDMARD start were conducted. Potential predictors of bDMARD/tsDMARD initiation were assessed in unadjusted analyses, age-sex-adjusted analyses, and multivariable analyses, separately for SpA and PsA. Covariates assessed included demographics, clinical characteristics, reported imaging findings, comorbidities, level of education, country of birth and family background. Multivariable analyses were performed in case of > 1 covariate with a *p*-value < 0.10 in the unadjusted models. In cases of significant collinearity (*r* > 0.3 with *p* < 0.05), only the covariate displaying the most robust association in the unadjusted model was incorporated into the multivariable model.

Proportional hazards (PH) assumptions for all models were examined. For dichotomous variables, the PH assumption was tested by visual examination of plots for log-minus-log function and follow-up time. For continuous variables, the PH assumption was tested by correlation of the partial residuals from cox regression analyses with the rank of follow-up time, and *r* > 0.3 with *p* < 0.05 was used as the cut off for exclusion.

A sensitivity analysis was conducted to evaluate potential interaction effects between CRP levels and the presence of bone marrow edema (BME) on MRI of the sacroiliac joints.

Sensitivity analyses with Cox regression restricted to patients with complete clinical data (i.e. psoriasis at diagnosis, subtypes of PsA, Psoriasis nail changes, CRP and ESR and clinical examination for patients with PsA; symptom duration, peripheral joint arthritis, MRI and the types of MRI findings (fatty lesions, BME and erosion at SIJ), HLA-B27, CRP and ESR for patients with SpA) were performed to assess the potential impact of missing data on the robustness of the findings.

In exploratory analyses, patients with PsA and SpA were divided into equal quartiles based on age at diagnosis, and this variable was modelled using the youngest quartile as reference.

### Prior conference presentation

A brief summary of a subset of the results of this study were published in the conference supplement for the 2024 EULAR congress in Vienna [17].

## Results

### Study population

A total of 188 patients with a new registered diagnosis of PsA and 148 patients with a new registered diagnosis of SpA during the study period were identified. Of these, 69 and 28 cases, respectively, were excluded after the review of medical records, as the diagnosis was not supported by available data according to the expert opinion of the rheumatologist.

Among the 119 patients with a validated diagnosis of PsA, the median symptom duration at the time-point of diagnosis was 17 months (interquartile range, IQR 9 - 36). The average age at diagnosis was of 49 years (standard deviation, SD 15.4), and 49% were males. (Table 1) The predominant phenotype was polyarthritis, in 69 patients, followed by asymmetric oligoarthritis (*n* = 31). A minority of cases, approximately 5%, exhibited a predominantly axial phenotype. The majority of patients with PsA had a previous diagnosis of psoriasis, and/or clinical psoriasis at diagnosis (Table 1). Forty-one patients initiated Methotrexate treatment at baseline, with an average dose of 18.4 mg/week. Typical X-ray findings at diagnosis were found in a minority of the patients (39.6%). Among patients with PsA, 94 (79%) fulfilled the CASPAR classification criteria. Baseline characteristics, i.e. clinical features at PsA diagnosis, comorbidities and sociodemographic variables are reported in Table 1. Substantial proportions in the PsA cohort had previously diagnosed comorbidities, with a CCI of ≥ 1 in 20%.

**Table 1:** Baseline characteristics of the patients with psoriasis arthritis

Characteristic	Statistics	**Missing data** n (%)
N	119	
Male sex	58 (48.7)	0
Age (years); mean (SD)	49 (15.4)	0
Symptom duration to diagnosis (months); median (IQR)	17 (9–36)	0
Smoking Current smokers Ever smokers	26 (28.3) 49 (52.1)	27 (22.7) 25 (21)
Laboratory parameters of inflammation CRP mg/L, median (IQR) Elevated CRP, n (%) ESR mm, median (IQR) Elevated ESR, n (%)	3.4 (1.4–9.6) 61 (52.1) 15 (7.0–29.3) 39 (43.2)	3 (2.5) 3 (2.5) 6 (5) 6 (5)
Subtype of PsA Polyarthritis Oligoarthritis Axial Other or undefined subtypes^#^	69 (57.5) 31 (25.8) 7 (5.8) 13 (10.8)	0
Psoriasis Family history for psoriasis Psoriasis before diagnosis Psoriasis at diagnosis Psoriatic nail changes at diagnosis	41 (34.5) 97 (81.5) 96 (80.7) 38 (32.2)	46 (38.7) 0 0 1 (0.8)
Clinical spondylitis	12 (10.1)	0
Clinical sacroiliitis	21 (17.6)	0
Clinical dactylitis	22 (18,5)	0
Clinical heel enthesitis	16 (13.4)	0
Complete clinical data*	108 (90.8)	0
Anterior uveitis, ever	13 (10.9)	73 (61.3)
Rheumatoid factor positive	5 (4.2)	14 (11.8)
X-Ray at diagnosis	97 (85.8)	7 (5.9)
X-Ray findings Erosive Joint space narrowing Proliferative Any of the above X-Ray changes	17 (17.3) 18 (18.0) 19 (19.4) 38 (39.6)	24 (20.2) 22 (18.5) 24 (20.2) 23 (19.3)
CASPAR criteria fulfilled	94 (79.0)	0
Education level ≤Lower secondary Upper Secondary / Short-cycle tertiary ≥Bachelor’s degree or equivalent	23 (19.3) 55 (46.2) 41 (34.5)	0
Country of birth Sweden Europe outside Sweden Rest of the world	87 (73.1) 17 (14.3) 15 (12.6)	0
Family background Both parents born in Sweden** Foreign family background***	72 (60.5) 47 (39.5)	0
Charlson’s comorbidity index ≥ 1	24 (20.2)	0
≥1 Pain related comorbidity	58 (48.7)	0
≥1 Psychiatric comorbidity	41 (34.5)	0
≥1 Other major comorbidity	52 (43.7)	0

Values are n (%) unless otherwise indicated

^#^DIP joint arthritis, arthritis mutilans, or undefined subtype of PsA

*Data on Psoriasis at diagnosis, subtypes of PsA, Psoriasis nail changes, CRP and ESR and clinical examination were available

**patient born in Sweden

***≥1 parent born abroad; patient born in Sweden or abroad

Among the120 patients with a validated diagnosis of SpA (mean age at diagnosis 38 years (SD 13); 64% males), the median symptom duration was 44 months (IQR 11–122). Of them 88 (73%) fulfilled the ASAS classification criteria. 78 patients (65%) fulfilled the ASAS classification criteria for axial SpA, 19 (16%) fulfilled the corresponding criteria for peripheral SpA, and 9 patients fulfilled both criteria sets. Baseline characteristics, i.e., clinical features at SpA diagnosis, history of anterior uveitis or inflammatory bowel diseases, level of formal education, categories of prevalent comorbidities, Charlson’s comorbidity index, country of birth and family background are reported in Table 2.

**Table 2:** Baseline characteristics of the patients with Spondyloarthritis

Characteristic at diagnosis	Statistics	Missing data n (%)
N	120	
Age (years); mean (SD)	38.2 (13.1)	0
Male sex	77 (64.2)	0
Symptom duration to diagnosis (months); median (IQR)	44 (11–122)	6 (5)
Smoking Current smoker Ever smoker	22 (18.3) 35 (38.9)	30 (25) 30 (25)
HLA-B27 positive	65 (70.7)	28 (23.3)
MRI SI-joints bone marrow edema fatty lesions erosion	107 (89.2) 68 (61.8) 48 (43.6) 46 (41.8)	3 (2.5) 10 (8.3) 10 (8.3) 10 (8.3)
Laboratory parameters of inflammation CRP mg/L, median (IQR) Elevated CRP * ESR mm, median (IQR) Elevated ESR *	3.4 (1.0–9.05) 61 (52.1) 12 (7.0–19.5) 37 (32.7)	3 (2.5) 3 (2.5) 7 (5.8) 7 (5.8)
Complete clinical data**	96 (80.0)	0
Current peripheral joint synovitis	13 (10.8)	0
Current psoriasis	5 (4.2)	10 (8.3)
Previous psoriasis	5 (4.2)	10 (8.3)
Inflammatory bowel disease ***	9 (8.3)	11 (9.2)
Anterior Uveitis ***	16 (13.3)	0
ASAS criteria for SpA fulfilled, any Axial SpA Peripheral SpA Both axial and peripheral SpA	88 (73.3) 78 (65.0) 19 (15.8) 9 (7.5)	0
Charlson’s comorbidity index ≥ 1	11 (9.1)	0
≥1 Pain related comorbidity	77 (64.2)	0
≥1 Psychiatric comorbidity	31 (25.8)	0
≥1 Other major comorbidity	38 (31.7)	0
Education level ≤Lower secondary Upper secondary / Short tertiary ≥Bachelor’s degree or equivalent	11 (9.2) 52 (43.3) 57 (47.5)	0
Country of birth Sweden Europe outside Sweden Rest of the world	92 (76.7) 15 (12.5) 13 (10.8)	0
Family background Both parents born in Sweden**** Foreign family background #	92 (76.7) 28 (23.3)	0

Values are n (%) unless otherwise indicated

*Above normal limit defined by the local laboratory (<3.0 mg/l for CRP and

**Data on symptom duration, peripheral joint arthritis, MRI and the types of MRI findings at SI-joint (bone marrow edema, fatty lesions, and erosions), HLA-B27, CRP and ESR were available

***Current or previous

*****patient born in Sweden

≥1 parent born abroad; patient born in Sweden or abroad

### Initiation of b/tsDMARD in patients with psoriatic arthritis

**Fig. 1:**
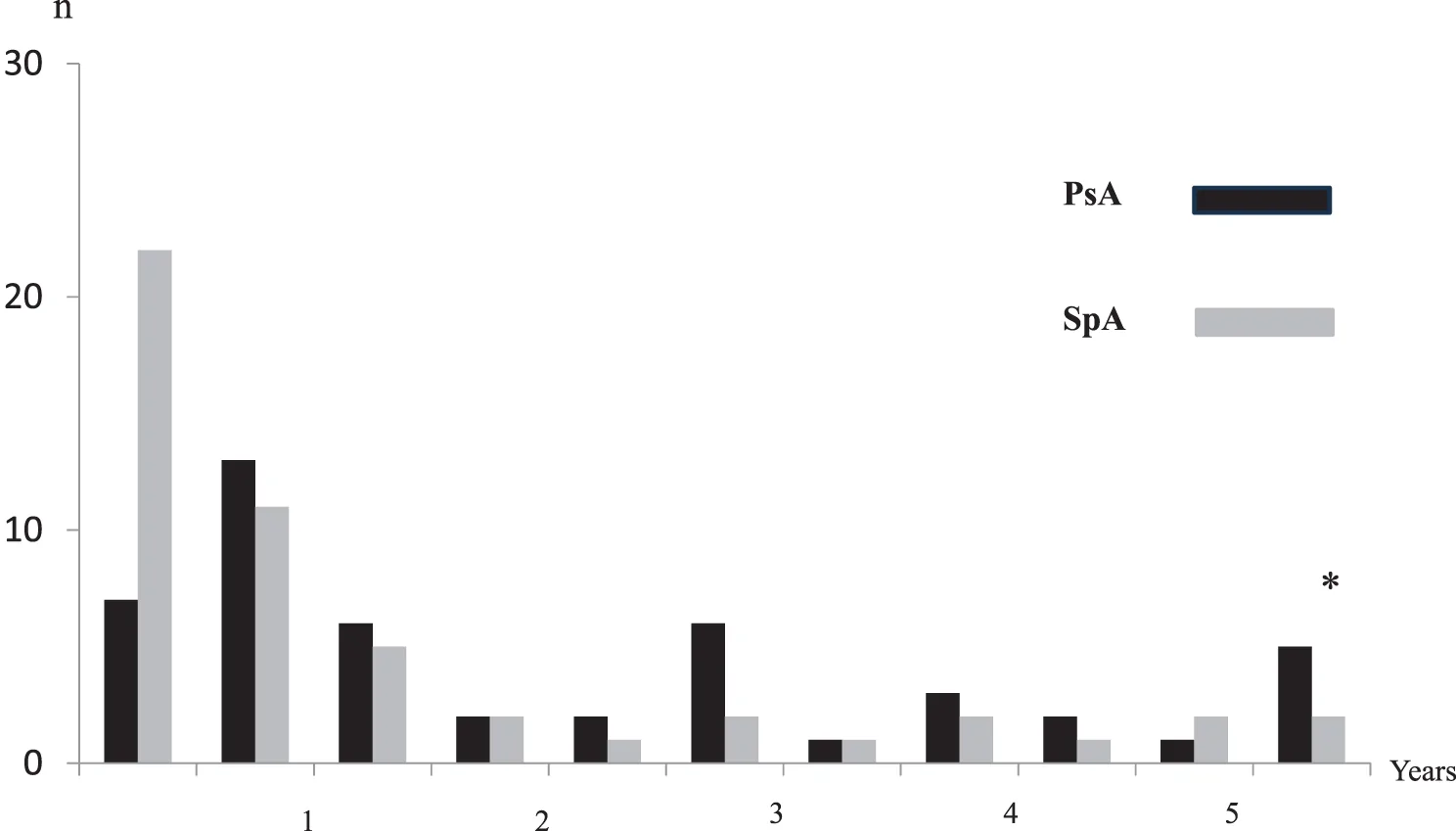
Number of patients with PsA or SpA initiating their first b/tsDMARD, by 6-month interval of time since PsA or SpA diagnosis. *This category includes all patients that started their first b/tsDMARD ≥ 5 years after diagnosis. b/tsDMARD, biologic/targeted synthetic disease-modifying antirheumatic drug

In this cohort, at time of PsA diagnosis, 4 patients were on treatment with a bDMARD (TNF-inhibitors in all cases), based on a prior diagnosis of psoriasis. These were excluded from the predictor analyses. Among the remaining, 48 patients (41.7%) started a first b/tsDMARD during the follow up, after a median time of 15 (IQR 8–38) months (Fig. 1). Two patients received a tsDMARD (apremilast and tofacitinib, respectively) without prior bDMARD therapy. For the remainder, the first bDMARD was in most cases a TNFi, except one who received ustekinumab (Supplementary Table 2).

### Initiation of b/tsDMARD in patients with spondyloarthritis

Two patients that were on treatment with infliximab at the time of SpA diagnosis, due to pre-existing inflammatory bowel diseases (IBD) were excluded from the predictor analyses. Among the remaining, 49 (41.5%) initiated treatment with a first bDMARD after diagnosis, with a median time from diagnosis to initiation of 7 months (IQR 3–16) (Fig. 1). No patient was prescribed a tsDMARD prior to first start of bDMARD. All patients were started on a TNF-inhibitor as the first bDMARD (Supplementary Table 2).

### Predictors of b/tsDMARD treatment in patients with psoriatic arthritis

An inverse relationship between the age at PsA diagnosis and the probability of initiating b/tsDMARD treatment was observed (HR per SD 0.74; 95% CI 0.55–1.00). There were no differences in time to initiation of first b/tsDMARD by sex, subtype of PsA, level of education, family background, country of birth or comorbidities in this cohort (Table 3). Furthermore, there were no associations with initiation of b/tsDMARDs for baseline laboratory findings (elevated CRP and ESR), radiographic joint damage or other disease manifestations (Table 3). The proportional hazards assumptions were assessed for all models and showed no significant violations.

**Table 3:** Predictors at diagnosis for the start of a first b/tsDMARD in patients with psoriasis arthritis – Cox regression

Variable in pat with PsA	HR (95% CI)
Male sex	0.93 (0.53–1.65)
Age (per SD)	**0.74 (0.55–1.00)**
Current smoker	0.92 (0.43–1.95)
Ever smoker	1.21 (063–2.30)
Elevated CRP*	0.99 (0.56–1.75)
Elevated ESR*	1.38 (0.76–2.50)
Erosion on X-Ray at diagnosis	1.54 (0.71–3.36)
JSN on X-Ray at diagnosis	1.09 (0.50–2.37)
Proliferative changes on X-Ray at diagnosis	0.99 (0.44–2.24)
Any X-Ray findings	1.08 (0.56–2.07)
Type of Arthritis Polyarthritis Oligoarthritis Axial Other/undefined subtypes	1 (ref) 0.77 (0.39–1.54) 0.55 (0.07–4.01) 1.19 (0.50–2.87)
Polyarthritis vs all other arthritis	1.18 (0.66–2.11)
Family history for Psoriasis	1.87 (0.88–4.00)
Psoriasis before diagnosis	0.59 (0.31–1.14)
Psoriasis at diagnosis	0.80 (0.41–1.56)
Psoriatic nail changes at diagnosis	1.18 (0.66–2.17)
Symptom duration	1.04 (0.97–1.12)
Anterior uveitis, ever	1.75 (0.69–4.42)
Clinical dactylitis	0.92 (0.43–1.96)
Clinical Heel enthesitis	1.04 (0.32–3.34)
Education level Low secondary Upper Secondary / Short-cycle tertiary Bachelor’s degree or equivalent	1 (ref) 0.83 (0.39–1.78) 0.92 (0.42–2.00)
Country of birth, n Sweden Europe outside Sweden Rest of the world	1 (ref) 0.68 (0.29–1.60) 0.38 (0.12–1.23)
Family background ≥1 parent born abroad, and subject born in Sweden or abroad	0.73 (0.40–1.34)
Charlson’s comorbidity index ≥ 1	0.90 (0.45–1.85)
≥1 Pain related comorbidity	1.23 (0.70–2.17)
≥1 Psychiatric comorbidity	1.19 (0.66–2.15)
≥1 Other major comorbidity	1.17 (0.66–2.10)

Numbers in bold indicate statistical significance (*p* < 0.05)

*Above normal limit defined by the local laboratory (3.0 mg/l for CRP and normal limit for age and sex for ESR)

### Predictors of bDMARD treatment in patients with spondyloarthritis

Patients with elevated CRP levels (HR 2.68; 95% CI 1.44–5.01), as well as those with evidence of BME on the MRI of the SIJ (HR 1.94; 95% CI 1.02–3.70), were more likely to receive bDMARD treatment, with similar results in age- and sex-adjusted models (Table 4). There was also a trend towards an association between peripheral arthritis at diagnosis and subsequent bDMARD treatment [hazard ratio (HR 2.11; 95% CI 0.97–4.59)]. No associations were observed between the initiation of bDMARDs and variables such as age, sex, level of education, family background, or country of birth or comorbidities in this patient cohort (Table 4). In the final multivariable model, elevated CRP and BME on the MRI of the SIJ at diagnosis were independent predictors for the initiation of bDMARD therapy (Table 4).

**Table 4:** Predictors at diagnosis for the start of a first bDMARD in patients with spondyloarthritis – Cox regression

Characteristic at diagnosis	Crude HR (CI 95%)	Age- & Sex-adjusted HR (CI 95%)	Multivariable HR (CI 95%)
Age (per SD)	0.88 (0.66–1.18)	N/A	NI
Male sex	1.04 (0.58–1.85)	N/A	NI
Current smoker	0.81 (0.39–1.71)	0.81 (0.37–1.76)	NI
Ever smoker	0.82 (0.43–1.58)	0.88 (0.43–1.78)	NI
HLA-B27 positivity	0.85 (0.43–1.71)	0.77 (0.35–1.68)	NI
MRI SI-joint bone marrow edema	**1.94 (1.02–3.70)**	**1.93 (1.01–3.67)**	**2.01 (1.04–3.90)**
MRI SI-joint fatty lesions	0.92 (0.51–1.65)	0.99 (0.54–1.80)	NI
MRI SI-joint erosion	1.30 (0.79–2.14)	1.65 (0.92–2.96)	NI
Elevated CRP*	**2.68 (1.44–5.01)**	**2.87 (1.52–5.41)**	**2.31 (1.21–4.40)**
Elevated ESR*	1.61 (0.90–2.90)	1.64 (0.91–2.97)	NI
Current Peripheral joint synovitis	2.10 (0.97–4.42)	2.11 (0.97–4.59)	1.53 (0.63–3.90)
Inflammatory bowel diseases**	0.84 (0.26–2.70)	0.79 (0.24–2.56)	NI
Anterior uveitis**	0.51 (0.18–1.41)	0.52 (0.18–1.44)	NI
Education ≤Lower secondary Upper secondary / Short tertiary ≥Bachelor’s degree or equivalent	1 (ref) 0.72 (0.29–1.80) 0.66 (0.27–1.63)	1 (ref) 0.75 (0.30–1.90) 0.67 (0.27–1.69)	NI
Charlson’s comorbidity index ≥ 1	1.14 (0.45–2.87)	1.47 (0.52–4.12)	NI
≥1 Pain related comorbidity	1.14 (0.63–2.07)	1.19 (0.65–2.19)	NI
≥1 Psychiatric related comorbidity	1.15 (0.62–2.12)	1.17 (0.62–2.18)	NI
≥1 Other relevant comorbidity	1.04 (0.57–1.89)	1.18 (0.62–2.26)	NI
Country of birth Sweden Europe outside Sweden Rest of the world	1 (ref) 0.61 (0.21–1.70) 1.66 (0.80–3.45)	1 (ref) 0.61 (0.22–1.73) 1.76 (0.84–3.69)	NI
Family background Foreign family background***	1.08 (0.57–2.03)	1.11 (0.59–2.11)	NI

Covariates demonstrating a *p*-value < 0.10 in the unadjusted models were eligible for incorporation into the multivariable analyses. In instances of significant collinearity (*r* > 0.3 with *p* < 0.05), only the covariate displaying the most robust association in the unadjusted model was included in the multivariable model

Numbers in bold indicate statistical significance (*p* < 0.05)

N/A: Not applicable. NI: Not included

*Above normal limit defined by the local laboratory (3.0 mg/l for CRP and normal limit for age and sex for ESR)

**Current or previous

***≥1 parent born abroad

The proportional hazards assumptions were assessed for all models and showed no significant violations.

We performed an additional analysis to assess potential interaction between elevated CRP levels and the presence of BME on MRI of the SIJ. No statistically significant interaction was observed (*p* = 0.14).

### Sensitivity analyses

In the sensitivity analyses including only patients with PsA with complete clinical data (108 cases, 91%), the results were in the same direction as in the main analysis, but there was no statistically significant association between age and the start of bDMARD.

Results were largely similar for patients with SpA in the sensitivity analyses including only those with complete clinical data (96 cases, 80%), although the association between BME of the SIJ on MRI and subsequent bDMARD initiation did not reach significance in this subset. (Supplementary Table 3 and 4).

### Exploratory prediction analysis

In exploratory analyses, when the patients were divided by age into 4 equal quartiles, we found that patients with PsA in the highest age category at diagnosis (62 years or older) had the lowest probability of being treated with b/tsDMARD in the future (HR 0.34; 95% CI 0.13–0.87 compared to the youngest category, ≤35 years). (Table 5). There was no such difference across quartiles of age at diagnosis of SpA (Table 5).

**Table 5:** B/tsDMARD start in relation to categories of age at diagnosis (quartiles) in patients with PsA and SpA – Cox regression

Age Categories for Patients with PsA	
Age category	HR (95% CI)
Quartile 1 (21.9–35.5 years)	1 (ref)
Quartile 2 (35.5–49.1 years)	0.78 (0.37–1.65)
Quartile 3 (49.1–62.6 years)	0.86 (0.41–1.78)
Quartile 4 (62.6–91.7 years)	**0.34 (0.13–0.87)**
**Age Categories for Patients with SpA**	
**Age category**	**HR (95% CI)**
Quartile 1 (18.9–28.6 years)	1 (ref)
Quartile 2 (28.6–34.4 years)	0.74 (0.33–1.65)
Quartile 3 (34.4–46.8 years)	0.96 (0.45–2.04)
Quartile 4 (46.8–76.7 years)	0.72 (0.32–1.61)

Numbers in bold indicate statistical significance (*p* < 0.05)

## Discussion

In this study, a similar proportion of patients with early SpA and early PsA initiated b/tsDMARD treatment (around 42%). However, the time to treatment initiation was shorter in SpA patients, and baseline predictors of b/tsDMARD therapy differed. In patients with PsA, there was no association with disease activity parameters, but we found a clear impact of age at diagnosis on subsequent start of b/tsDMARD treatment. Older patients with PsA were significantly less likely to begin treatment with b/tsDMARDs, although the association was borderline and did not reach statistical significance in the complete case sensitivity analysis. Further analysis indicated that this mainly applied to the oldest quartile, those aged > 62 years. Interestingly, this inverse association between age and b/tsDMARD treatment initiation in PsA did not appear to be explained by differences in level of education, or comorbidities, as these had no significant impact on treatment initiation. By contrast, in patients with SpA, features indicating active disease at baseline (elevated CRP and presence of BME on MRI of SIJ) predicted subsequent treatment with bDMARDs. For example, patients with elevated CRP at SpA diagnosis were more than twice as likely to receive bDMARD treatment later during the disease course. No differences by sex were observed, and there were no indications of inequality related to ethnicity or educational background.

Prior studies indicate that multimorbidity, which is common in older populations, might contribute to the decreased commencement of bDMARD therapy in patients with RA [18]. However, in the present analysis of patients with PsA we did not find any such association for the Charlson comorbidity index, based on prevalent comorbidities. Similar findings regarding age, comorbidities and bDMARD treatment were observed in our recently published study on predictors for start of bDMARDs in patients with early RA [12]. It is well known that patients with PsA have an increased prevalence of many comorbidities compared to the general population [19] and to patients with psoriasis alone [20], and a substantial proportion of patients with PsA in this study suffered from comorbidities. However, there were no significant associations between b/tsDMARD initiation and the investigated groups of comorbidities in patients with PsA.

Numerous studies corroborate the observation that early initiation of bDMARDs is less common among elderly individuals when considering arthritis diseases overall [21]. This pattern could be partially attributed to apprehensions regarding adverse events, particularly the risk of infections, among the elderly [22]. Furthermore, early bDMARD treatment in younger patients offers significant potential benefits, including prevention of disease progression, preserved joint function, and improved long-term quality of life.

By contrast, in patients with SpA age does not seem to be a determinant of early start of bDMARDs. One plausible explanation for this distinction could be the demographic differences between the two cohorts, with SpA patients presenting at a younger age compared to those with PsA. Moreover, prognostication in early SpA may be more straightforward compared to PsA, with several standard biomarkers that are considered to be useful for treatment decisions. The final multivariable analysis revealed that elevated CRP levels and BME on MRI of the SIJ independently predict the initiation of bDMARD therapy. On the other hand, as data on disease activity scores were not available, we cannot exclude that the underlying treatment decisions were driven by the clinical disease activity. The findings of this study are consistent with management according to the latest ASAS-EULAR guidelines, which recommend that b/tsDMARDs should be considered in patients with elevated CRP and/or positive MRI of the SI joints that fail standard treatment and have high disease activity [6].

The limited effectiveness of conventional synthetic DMARDs (csDMARDs) for axial inflammation has led to earlier use of bDMARDs in SpA than in PsA. Studies indicate that csDMARDs like methotrexate often fail to address the needs of patients with axial involvement, resulting in a quicker shift to bDMARDs for SpA management [23].

Management of PsA presents major challenges in clinical practice compared to that of RA, which benefits from the availability of disease specific autoantibodies and longer experience with bDMARD treatment. Furthermore, in the early stages PsA may present with joint tenderness before obvious signs of arthritis appear. These factors, together with the less structured diagnostic process in PsA compared to RA and SpA, may contribute to the limited available data regarding predictors at the time of diagnosis for initiating bDMARDs in patients with PsA.

There are several limitations inherent in our study that need to be acknowledged. First, our sample size was relatively small, which may have limited our ability to detect weaker associations. We cannot exclude that additional associations with b/tsDMARD initiation could have been captured in larger samples. Second, the involvement of different physicians in the assessment of patients could introduce variability in evaluations and decision making. However, this potential variability might be balanced out by the consistent follow-up provided by well-coordinated teams of rheumatologists and healthcare professionals within our single-center study setting. Inclusion was based on a validated diagnosis on retrospective review by a expert rheumatologist. Most, but not all patients fulfilled established classification criteria for PsA and SpA. Lack of fulfillment of such criteria may partly be due to inconsistent reporting of relevant data in the medical records. Third, the identification of comorbidities relied solely on registered diagnostic codes without validation. Fourth, data on findings from MRI of SIJ and radiographs of peripheral joints were based on derived from radiologists’ reports on assessment in clinical practice, and not evaluated using standardized scoring methods (i.e. ASAS definitions on sacroiliitis and the Sharp-van der Heijde modified scoring method for PsA, respectively). Furthermore, 12% of MRIs of SIJ were performed outside the time window of 6 months before/after diagnosis. The lack of standard time points and of standard assessments for imaging could limit our ability to detect true associations for imaging would not be expected to introduce systematic bias. Additionally, other relevant data on lifestyle factors like body mass index and alcohol use, severity of psoriasis, disease activity scores for SpA and PsA, family history of SpA, other medication use and circulating biomarkers or genetics from analyses of stored blood samples were not available. Therefore, we cannot exclude that some of these factors may be associated with future start of bDMARD therapy. Furthermore, patients who migrated to another region and initiated a b/tsDMARD but not included in the SRQ would not have data on the outcome, possibly introducing bias. The single-center setting may also limit generalizability. It is important to recognize that our findings may not be generalizable to countries with healthcare systems differing from Sweden’s model, where equal healthcare access is provided to all citizens regardless of socioeconomic status. Therefore, caution should be exercised when extrapolating our results to settings with different healthcare structures, particularly those reliant on private insurance or providers. Finally, as multiple comparison correction was not applied, analyses should be regarded as exploratory.

There are several strengths that contribute to the validity of this study. Firstly, the sample of patients included is representative of clinical practice and resource utilization within our geographic region, enhancing the applicability of our findings to real-world scenarios. Moreover, all data were meticulously collected from reliable registry sources, supplemented by comprehensive medical record reviews. The SRQ register has demonstrated high coverage and data accuracy regarding bDMARD exposure in SpA/PsA, further underlining the reliability of our findings [9].

## Conclusion

In this study, older individuals diagnosed with PsA were less likely to start biologic treatment early on, while no significant associations were found between treatment initiation and sex, disease subtype and baseline disease characteristics. By contrast, among SpA patients, elevated CRP levels and evidence of active sacroiliitis on MRI were linked to early biologic treatment initiation, although the relative impact of clinical disease activity could not be evaluated. These findings underscore the importance of objective measures of inflammation for treatment decisions in early SpA, and the challenges in evaluating disease severity as a basis for prognosis in early PsA. Sex, country of origin or level of education and ethnicity were not associated with bDMARD initiation, suggesting no major inequity in access to such treatment in this patient group.

## Electronic supplementary material

Below is the link to the electronic supplementary material.

Supplementary Material 1

Supplementary Material 2

Supplementary Material 3

## Data Availability

The datasets generated and/or analyzed during the current study are not publicly available due to Swedish legislation, but a limited set of data that support the main analyses is available from the corresponding author on reasonable request.
