# Supplementary material for: Predictors at diagnosis for initiation of biologic or targeted synthetic disease-modifying anti-rheumatic drugs in patients with spondyloarthritis and psoriatic arthritis
Source: BMC Rheumatol. 2026 Sep 29;10:75. doi: 10.1186/s41927-026-00698-w (PMC13621707; doi:10.1186/s41927-026-00698-w)
Supplement: Supplementary file 1 [file 41927_2026_698_MOESM1_ESM.docx]

**Supplementary Table 1 .** Comorbidities based on ICD-10 code

| **Comorbidity** | **ICD-10 code** | **SpA n (%)** | **PsA n (%)** |
| --- | --- | --- | --- |
| *Pain related comorbidities* | | |  |
| Spinal stenosis | M48.0, M48.8 | 2 (1.7) | 2 (1.7) |
| Vertebral compression fracture | M48.4, M48.5, M49.5 | 0 | 0 |
| Degenerative disc disease with nerve pain, including sciatica | M50.1, M54.1, M54.2, M54.3, M54.4 | 36 (30.0) | 22 (18.5) |
| Osteoarthritis | M15-M19 | 9 (7.5) | 28 (23.5) |
| Gastritis/Peptic/Gastric ulcer/Esophagitis/Dyspepsia | K20-21, K25-K27, K29-K30 | 17 (14.2) | 17 (14.3) |
| Neuroborreliosis | G01*A69.2 | 0 | 2 (1.7) |
| Neuralgia (Zoster/trigeminal/Non specified) | G50.0, G53.0, M79.2 | 0 | 1 (0.8) |
| IBS | K58.1, K58.2, K58.3, K58.8 | 0 | 0 |
| Nonspecific pain, Generalized pain | R52.1, R52.2, R52.9 F454 | 58 (48.3) | 52 (43.7) |
| Fibromyalgia | M.79.7 | 8 (6.7) | 14 (11.8) |
| ≥1 pain related comorbidity |  | 77 (64.2) | 58 (48.7) |
| *Psychiatric comorbidities* | | |  |
| Depression | F31-F34 | 15 (12.5) | 19 (16.0) |
| Anxiety/Psychological stress | F41, F43, F06.4 | 25 (20.8) | 34 (28.6) |
| Diagnoses related to alcohol abuse | F10 | 4 (3.3) | 5 (4.2) |
| ≥1 psychiatric comorbidity |  | 31 (25.8) | 41 (34.5) |
| *Other relevant comorbidities* | | |  |
| Kidney failure | N17, N18.2, N18.3, N18.4, N18.5, N18.9, N19.9 | 0 | 2 (1.7) |
| Thyroid disease | E03, E05, E06 | 8 (6.7) | 7 (5.9) |
| Ischemic heart disease | I20-I25 | 3 (2.5) | 9 (7.6) |
| Chronic heart failure | I50 | 0 | 3 (2.5) |
| Cerebrovascular disease | I60-I69 | 1 (0.8) | 7 (5.9) |
| Peripheral vascular disease | I70-I74, I77, I79 | 2 (1.7) | 2 (1.7) |
| Chronic diseases of the airways | J40-J47, J60-J64, J66-J67, J82, J84 | 14 (11.7) | 21 (17.6) |
| Obesity | E66 | 3 (2.5) | 14 (11.8) |
| Liver disease | K70-K77 | 0 | 3 (2.5) |
| Diabetes | E10-E14 | 4 (3.3) | 12 (10.1) |
| Malignancy | C00-C97, D00-D09, D37-D48 | 11 (9.2) | 16 (13.4) |
| IBD | K50-K51 | 9 (7.5) | 0 |
| Multiple sclerosis | G35.9 | 0 | 0 |
| ≥1 other relevant comorbidity |  | 46 (38.3) | 52 (43.7) |

**Supplementary Table 2:** Distribution of first b/tsDMARD treatment for patients with SpA and PsA diagnosed in the period 2012-2016

| **SpA** | | |
| --- | --- | --- |
| **Type of bDMARD** | | **n (%)** |
| Adalimumab | | 22 (44.9) |
| Etanercept | | 14 (28.6) |
| Certolizumab pegol | | 2 (4.1) |
| Infliximab | | 8 (16.3) |
| Golimumab | | 3 (6.1) |
| ***Total*** | | ***49*** |
| **PsA** | | |
| **Type of b/tsDMARD** | **n (%)** | |
| Adalimumab | 14 (30.4) | |
| Etanercept | 24 (52.2) | |
| Certolizumab pegol | 1 (2.2) | |
| Infliximab | 5 (10.9) | |
| Golimumab | 1 (2.2) | |
| Ustekinumab | 1 (2.2) | |
| Apremilast | 1 (2.2) | |
| Tofacitinib | 1 (2.2) | |
| ***Total*** | ***48*** | |
