# Supplementary material for: Predictors at diagnosis for initiation of biologic or targeted synthetic disease-modifying anti-rheumatic drugs in patients with spondyloarthritis and psoriatic arthritis
Source: BMC Rheumatol. 2026 Sep 29;10:75. doi: 10.1186/s41927-026-00698-w (PMC13621707; doi:10.1186/s41927-026-00698-w)
Supplement: Supplementary file 2 [file 41927_2026_698_MOESM2_ESM.docx]

**Supplementary Table 3: Predictors at diagnosis for the start of a first b/tsDMARD in patients with Psoriatic arthritis having complete clinical data– Cox regression**

| **Characteristic** | **HR (95% CI)** |
| --- | --- |
| Male sex | 0.97 (0.54 – 1.73) |
| Age (per SD) | 0.78 (0.57 – 1.06) |
| Current smoker | 0.90 (0.42 – 1.93) |
| Ever smoker | 1.20 (0.62 – 2.32) |
| Elevated CRP* | 1.00 (0.56 – 1.78) |
| Elevated ESR* | 1.36 (0.74 – 2.49) |
| Erosion on X-Ray at diagnosis | 1.64 (0.90 – 2.96) |
| JSN on X-Ray at diagnosis | 1.26 (0.30 – 5.18) |
| Proliferative changes on X-Ray at diagnosis | 1.01 (0.44 – 2.29) |
| Any X-Ray findings | 0.81 (0.38 – 1.74) |
| Type of Arthritis  Polyarthritis  Oligoarthritis  Axial  Other/undefined subtypes | 1 (ref)  0.86 (0.43 – 1.72)  0.79 (0.11 – 5.84)  1.32 (0.54 – 3.18) |
| Polyarthritis vs all other arthritis | 1.04 (0.57 – 1.88) |
| Family history for Psoriasis | 1.79 (0.82 – 3.90) |
| Psoriasis before diagnosis | 0.59 (0.31 – 1.15) |
| Psoriasis at diagnosis | 0.88 (0.45 – 1.73) |
| Psoriatic nail changes at diagnosis | 1.23 (0.68 – 2.23) |
| Symptom duration | 1.04 (0.96 – 1.13) |
| Anterior uveitis, ever** | 1.10 (0.42 – 2.89) |
| Clinical dactylitis | 0.99 (0.48 – 2.06) |
| Clinical Heel enthesitis | 1.14 (0.55 – 2.40) |
| Education level  Low secondary  Upper Secondary / Short-cycle tertiary  Bachelor’s degree or equivalent | 1 (ref)  0.94 (0.42 – 2.08)  1.09 (0.49 – 2.43) |
| Country of birth  Sweden  Europe outside Sweden  Rest of the world | 1 (ref)  0.74 (0.29 – 1.88)  0.29 (0.69 – 1.18) |
| Family background  ≥1 parent born abroad, and patient born in Sweden or abroad | 0.72 (0.38 – 1.34) |
| Charlson’s comorbidity index ≥1 | 0.82 (0.38 – 1.76) |
| ≥ 1 Pain related comorbidity | 1.21 (0.68 – 2.17) |
| ≥ 1 Psychiatric comorbidity | 1.11 (0.61 – 2.02) |
| ≥ 1 Other major comorbidity | 1.10 (0.62 – 1.97) |
| * Above normal limit defined by the local laboratory (<3.0 mg/l for CRP and <normal limit for age and sex for ESR)  ** Current or previous | |
