# Supplementary material for: Predictors at diagnosis for initiation of biologic or targeted synthetic disease-modifying anti-rheumatic drugs in patients with spondyloarthritis and psoriatic arthritis
Source: BMC Rheumatol. 2026 Sep 29;10:75. doi: 10.1186/s41927-026-00698-w (PMC13621707; doi:10.1186/s41927-026-00698-w)
Supplement: Supplementary file 3 [file 41927_2026_698_MOESM3_ESM.docx]

**Supplementary Table 4: Predictors at diagnosis for the start of a first bDMARD in patients with spondyloarthritis having complete clinical data – Cox regression**

| **Characteristic at diagnosis** | **Crude HR (95% CI)** | **Age- & Sex-adjusted**  **HR (95% CI)** |
| --- | --- | --- |
| Age (per SD) | 0.90 (0.61 – 1.32) | N/A |
| Male sex | 1.62 (0.80 – 3.25) | N/A |
| Current smoker | 1.19 (0.55 – 2.57) | 1.10 (0.50 – 2.44) |
| Ever smoker | 1.31 (0.65 – 2.64) | 1.31 (0.62 – 2.74) |
| HLA-B27 positivity | 1.32 (0.59 – 2.96) | 1.12 (0.45 – 2.77) |
| MRI SI-joint bone marrow edema | 1.81 (0.91 – 3.56) | 1.73 (0.87 – 3.43) |
| MRI SI-joint fatty lesions | 0.85 (0.45 – 1.62) | 0.88 (0.46 – 1.68) |
| MRI SI-joint erosion | 1.50 (0.80 – 2.81) | 1.58 (0.83 – 3.01) |
| Elevated CRP* | **2.94 (1.46 – 5.92)** | **2.86 (1.41 – 5.80)** |
| Elevated ESR* | 1.43 (0.74 – 2.75) | 1.34 (0.69 – 2.59) |
| Current Peripheral joint synovitis | 1.71 (0.67 – 4.38) | 2.00 (0.76 – 5.25) |
| Inflammatory bowel diseases** | 0.86 (0.27 – 2.82) | 0.91 (0.28 – 2.99) |
| Anterior uveitis** | 0.65 (0.20 – 2.12) | 0.61 (0.19 – 2.02) |
| Education  ≤ Lower secondary  Upper secondary / Short tertiary  ≥ Bachelor’s degree or equivalent | 1 (ref)  0.63 (0.21 – 1.88)  0.50 (0.17 – 1.47) | 1 (ref)  0.77 (0.25 – 2.40)  0.59 (0.19 – 1.82) |
| Charlson’s comorbidity index ≥1 | 0.57 (0.14 – 2.36) | 0.55 (0.12 – 2.59) |
| ≥ 1 Pain related comorbidity | 1.13 (0.58 – 2.20) | 1.20 (0.61 – 2.36) |
| ≥ 1 Psychiatric related comorbidity | 1.24 (0.63 – 2.44) | 1.33 (0.67 – 2.65) |
| ≥ 1 Other relevant comorbidity | 0.83 (0.42 – 1.63) | 0.94 (0.45 – 1.95) |
| Country of birth  Sweden  Europe outside Sweden  Rest of the world | 1 (ref)  0.84 (0.29 – 2.39)  1.68 (0.73 – 3.84) | 1 (ref)  0.89 (0.31 – 2.53)  1.81 (0.78 – 4.22) |
| Family background  Foreign family background*** | 1.23 (0.61 – 2.47) | 1.31 (0.65 – 2.64) |
| N/A: Not applicable  * Above normal limit defined by the local laboratory (<3.0 mg/l for CRP and <normal limit for age and sex for ESR)  ** Current or previous  ***Patient born in Sweden | | |
